# Targeting iron metabolism in cancer therapy

**DOI:** 10.7150/thno.59092

**Published:** 2021-07-25

**Authors:** Michael Morales, Xiang Xue

**Affiliations:** Department of Biochemistry and Molecular Biology, University of New Mexico Health Sciences Center, Albuquerque, NM 87131

**Keywords:** Iron metabolism, Cancer, Therapy, Chelation, Ferroptosis

## Abstract

Iron is a critical component of many cellular functions including DNA replication and repair, and it is essential for cell vitality. As an essential element, iron is critical for maintaining human health. However, excess iron can be highly toxic, resulting in oxidative DNA damage. Many studies have observed significant associations between iron and cancer, and the association appears to be more than just coincidental. The chief characteristic of cancers, hyper-proliferation, makes them even more dependent on iron than normal cells. Cancer therapeutics are becoming as diverse as the disease itself. Targeting iron metabolism in cancer cells is an emerging, formidable field of therapeutics. It is a strategy that is highly diverse with regard to specific targets and the various ways to reach them. This review will discuss the importance of iron metabolism in cancer and highlight the ways in which it is being explored as the medicine of tomorrow.

## Introduction

Iron is essential for cell vitality. It is found in proteins that perform a variety of functions including biomolecule synthesis, oxygen transport and homeostasis, and respiration 1. Iron is a critical component of many proteins involved in nucleic acid metabolism and repair, as well as cell cycle progression 2. Because iron is an integral component of anatomy and physiology and its bioavailability is scarce, iron stores are tightly regulated within the body in order to ensure conservation and mitigate toxicity 3.

The oxidation-reduction (redox) ability of iron is at the heart of its importance as a handler of oxygen and electrons, but it is in this same role that it harbors its dangers 4. Iron is able to easily interconvert between the ferrous state (Iron [II]) and ferric state (Iron [III]) and may exist in a wider range of oxidation states 4. In cellular metabolism, iron largely draws its negative effects from the reduction of oxygen. Due to oxygen's atomic nature, its reduction must proceed in a stepwise fashion of individual electron additions and reactive intermediates 5. During this process, the Fenton reaction can occur between ferrous iron and hydrogen peroxide to generate the highly reactive hydroxyl radical 5. Oxygen reduction intermediates are known as reactive oxygen species (ROS) and have been linked to lipid, protein, nucleic acid, and various signaling pathway damage 6. As such, iron has become a key target of interest in the progression and treatment of diseases including cancer. This review will discuss aspects of the therapeutic potential of iron metabolism for cancer. First, we will present a brief overview of the role of iron in the body and discuss aspects of the therapeutic potential of iron for the treatment of cancer.

## Cellular Metabolism of Iron

### Iron Absorption and Recycling

**Figure 1:**
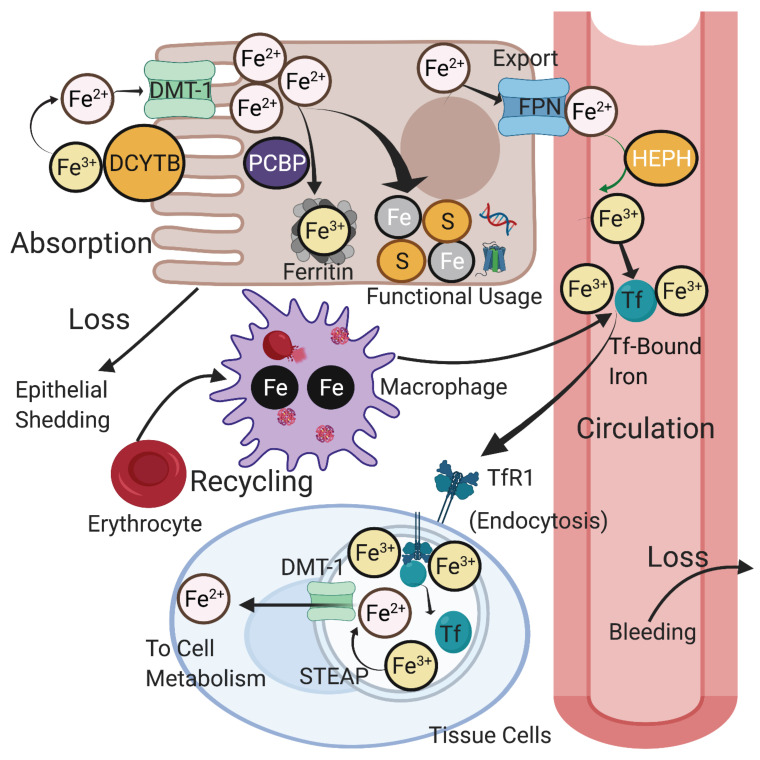
** Iron absorption and recycling.** Non-heme iron is absorbed into enterocytes by DMT-1 after reduction from Fe (III) to Fe(II) by DcytB. Iron is carried by chaperones such as PCBPs to sites for storage in ferritin or for functional usage in cellular proteins and metabolism. Iron can be exported through FPN and subsequently re-oxidized by HEPH to Fe (III). Most circulating iron is carried by Tf and delivered to various tissues via its receptor TfR1 through receptor-mediated endocytosis. Tf and ferric iron dissociate in the endosome, after which the ferric iron is reduced to ferrous iron by STEAP proteins and enters the cytosol. Circulating iron is mainly derived from phagocytosis in senescent red blood cells, a process mediated by macrophages. Iron loss from the body occurs regularly through tissue loss such as epithelial shedding and blood loss. DMT-1: divalent metal transporter 1; DcytB: duodenal cytochrome B; PCBP: poly(rC)-binding protein; FPN: ferroportin; HEPH: hephaestin; STEAP: six transmembrane epithelial antigen of the prostate; Tf: transferrin; TfR1: transferrin receptor 1. Created with BioRender.com

In a standard diet, inorganic iron (Fe^3+^) from plant origin accounts for 80-90%, whereas the remaining 10% is heme iron (Fe^2+^) associated with meat intake 7. Ingested inorganic iron must first be reduced to its ferrous form for solubility and absorption by enterocytes. Reduction of iron is achieved through ferrireductases, specifically duodenal cytochrome B (DcytB), and potentially in combination with other ferrireductases 8 (**Figure 1**). Ferrous iron is imported into enterocytes through divalent metal transporter 1 (DMT1) on the apical membrane 9. Heme iron and ferritin are also absorbed by enterocytes, but they require a slightly different path from inorganic iron absorption in order to release iron from their organic containers. Nevertheless, heme, ferritin, and inorganic iron are all able to be effectively incorporated into cells 8. In fact, heme iron can be more effectively absorbed and incorporated into the body than non-heme iron and may contribute up to 50% of the iron absorption from diet 10. Once in the cell, iron can be used for a number of functions depending on the cellular and systemic conditions at hand, which we will present.

Within enterocytes, redox-active iron is transiently collected in the labile iron pool as it passes from its entry stage into the greater cell metabolism 11. Chaperone proteins including Poly(rC)-binding proteins (PCBPs) and glutaredoxin-BolA-like protein complexes direct iron to various sites in the cell 12. Due to its redox capabilities, iron is utilized in the mitochondria for various processes as a part of prosthetic groups such as iron-sulfur clusters and porphyrin bound iron, or as an ion. Within these groups, iron functions in various electron transfer reactions such as within the tri-carboxylic acid/citric acid/Krebs cycle, the mitochondrial respiratory chain, molecular biosynthesis, and signaling 13. Within the nucleus of cells, iron modulates the regulation and function of various proteins involved in the replication and repair of DNA, including DNA helicase and the various polymerases 14. Iron is also critical for the synthesis of deoxyribonucleotides as a part of the redox chemistry-heavy ribonucleotide reductase 15. The general trend of cellular iron utilization by various proteins and reactions leverages the role of iron as a potent facilitator of electron transfer.

When not incorporated into functional purposes, iron can be safely stored within the cell. Ferritin is a protein that both oxidizes ferrous iron and retains the ferric form for stable storage 16. Depending on the iron needs of the cell and larger systems, iron may be released through ferroportin (FPN), the highly regulated and sole known exporter of iron in cells 17. Iron in systemic circulation may be picked up by other cells through the use of the iron transport protein transferrin (Tf) 18. The process of erythropoiesis is heavily dependent on systemic iron circulation due to the synthesis of hemoglobin in the red blood cells and functions in accordance with several homeostatic regulators that will be discussed later 19. Iron is also known to play an important role across the immune system in both myeloid and lymphoid cells. Iron can be used to generate anti-pathogenic compounds such as in neutrophils and eosinophils, and it can also be used to direct the development, differentiation, and polarization of T-cells and macrophages 20.

When ferrous iron leaves the intestinal epithelial cell via FPN, it is readily oxidized to its ferric form by the ferroxidase hephaestin (HEPH) 21 (**Figure 1**). In the circulation, two ferric ions load to the “empty” apo-Tf to form Holo-Tf, which carries the redox inactive iron to a transferrin receptor (TfR1) on a target cell in order to initiate receptor mediated endocytosis 18. In tissue cells, ferric iron is dependent on ferrireductase six transmembrane epithelial antigen of the prostate (STEAP) proteins to form ferrous iron. The ferrous iron is then exported out of the endosome and into the cytosol of the cell via DMT1 18, 22. This process is dependent on the controlled acidity of the endolysosomes, which must remain in a state of low (acidic) pH in order to release iron; disruption of this acidity can prevent the release of vital iron into the cytosol 23.

As a form of conservation, iron is recycled from the breakdown of old proteins in order to be repurposed. Most of the iron that is repurposed comes from senescent red blood cells, which are degraded by macrophages. Iron from the degraded hemoglobin is eventually detached and either stored within the macrophage or released through FPN 24. No regulated form of excretion is known, but iron turnover occurs regularly through blood loss and epithelial exfoliation 25. Iron turnover rates are not universal but vary by person, with annual rates ranging from as low as 2% to as high as 95% 26. Pre-menopausal women commonly have higher rates of iron loss due to menstruation 26.

### Iron Homeostasis

**Figure 2:**
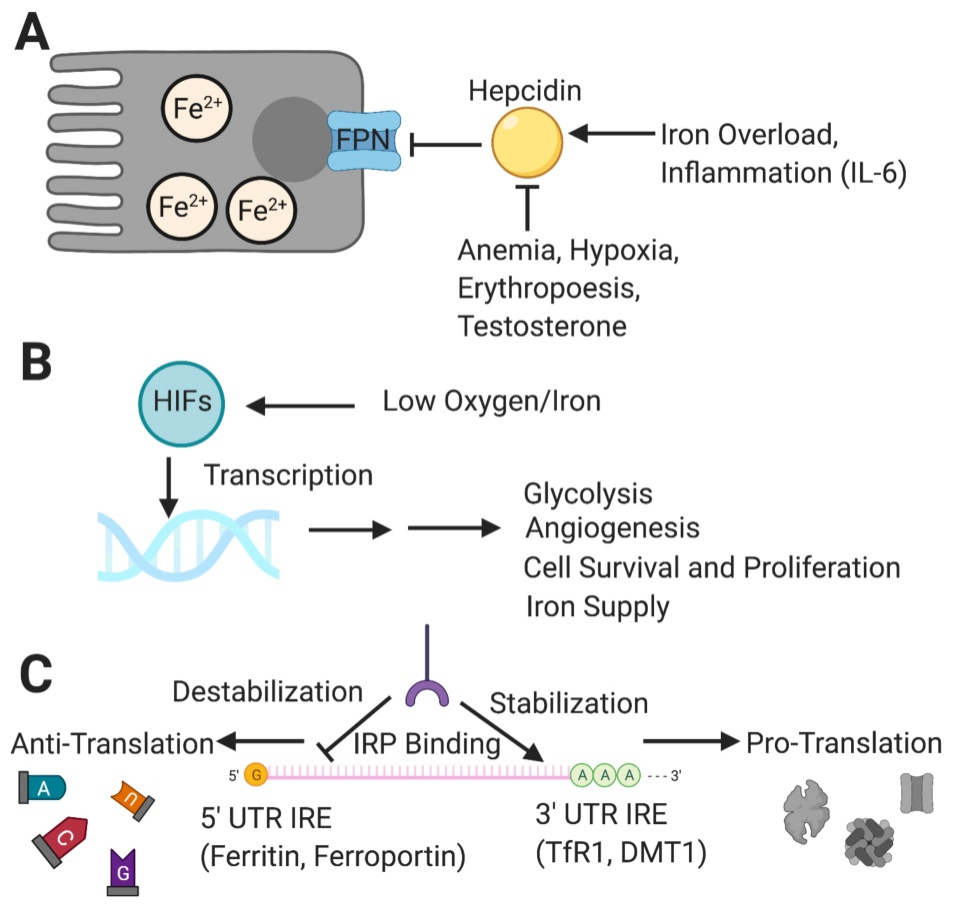
** Major regulators of iron homeostasis include hepcidin, HIFs and IRP/IRE systems. [A]** Hepcidin naturally limits the amount of iron efflux from cells and is inhibited by conditions such as anemia, hypoxia, increased testosterone, and increased erythropoiesis, while being upregulated by systemic iron overload and inflammation. [**B**] HIFs respond to low oxygen and iron levels and transcribe genes to help cells adapt to perceived environmental deficiencies for a more sustainable metabolism and long-term survival; this results in short- and long-term changes including increases in glycolysis, angiogenesis, iron supplies, and ultimately cell vitality. [**C**] IRPs control gene translation through binding of IREs on mRNA transcripts for iron metabolism-related proteins, either promoting translation through 3' UTR binding-dependent stabilization (e.g., TfR1, DMT1), or inhibiting translation through 5' UTR binding that results in eventual degradation (e.g., Ferritin, FPN). HIF: hypoxia-inducible factor; IRP: iron response protein; IRE: iron response element; mRNA: messenger RNA; UTR: untranslated region. Created with BioRender.com

To mitigate iron toxicity from overaccumulation, cells and organs must carefully coordinate the regulation of iron. This can occur at the level of transcription, translation, or post-translation of functional proteins 27. The regulation of iron homeostasis is centered on the control of iron circulation and involves FPN and its chief regulator, hepcidin 28 (**Figure 2A**). This peptide hormone is responsible for preventing iron overload in the body by inhibiting iron transport through binding to the exporter FPN, ultimately leading to FPN degradation 29. Hepcidin itself is regulated by several other molecules that either increase or decrease its production 29. In addition to high iron levels, inflammatory signaling molecules such as interleukin-6 (IL-6) will increase the production of hepcidin, while conditions of anemia and hypoxia, increased erythropoiesis and testosterone will inhibit the pathway 29. One way to summarize the role of hepcidin is that it prevents systemic iron overload by keeping iron within cells. Another way of looking at the function of hepcidin is that it increases the amount of iron in cells by preventing its escape.

In addition to hepcidin, hypoxia is critical for regulating the transcription of many other iron metabolic genes including DMT1, TfR1 and FPN (**Figure 2B**). In general, the condition of hypoxia is a more overarching control, for it has a more expansive genetic effector domain that it exerts control over through the use of a group of transcription factors known as hypoxia-inducible factors (HIFs) 30. Among the many downstream targets of HIFs are genes for glycolysis, angiogenesis, cell proliferation, and cell migration 31. HIFs exert potent inhibitory control over hepcidin levels, even more so than the upregulating effect of inflammation 32. HIF-1α and HIF-2α are the two most critical HIF isoforms regulating cellular metabolism. While there exists a degree of overlap between targets of the two major isoforms, HIF-1α appears to exert a stronger effect during the earlier stages of hypoxia and tends to target genes that shift cellular metabolism towards glycolysis. On the other hand, HIF-2α appears to be the more dominantly active isoform during a prolonged hypoxic response 33-35. The majority of HIF-2a target genes are involved in modulation of tumor cells and the microenvironment resulting in a shift toward long term growth. HIF targets include several genes affecting iron metabolism. The significance of these functions will be discussed further in the following sections. In summary, HIFs ultimately work to increase a cell or system's capacity for sustained metabolism.

Iron response proteins (IRPs) are another component of iron homeostasis, acting on the iron response elements (IREs) of messenger-RNA (mRNA) untranslated regions (UTRs) 36 (**Figure 2C**). As suggested by their names, their function changes based on the levels of iron, which they are able to sense. First studied for their effects on ferritin and TfR1, IRP-IRE interactions may either stabilize or destabilize mRNA transcripts depending on the binding site, and the effects vary by genes and depend on the iron needs of the cell 37.

## Iron and Cancer

As previously mentioned, there is an indisputable need for iron in the body. However, excess iron accumulation can be highly toxic. Dysregulated iron homeostasis due to hereditary and lifestyle factors may lead to increased risk of cancer. Cancer cells are characterized by rapid proliferation, which consequently demands a greater amount of iron and results in dysregulated levels of key proteins involved in iron metabolism 38, 39. Here we will describe these two aspects in details below.

### Hereditary and Lifestyle Risks

Excessive iron intake is associated with various cancers to differing degrees; some cancers show strong association while others may only have a potential link 40. According to a meta-analysis by Fonseca-Nunes et al., the body of epidemiological data surrounding the risk for colorectal cancer from dietary iron overload appears to carry the strongest implications with regard to iron consumption and cancer, while studies of other cancers such as breast, esophageal, gastric, and lung report a range of association from moderately suggestive to inconclusive due to varying results of association and statistical significance 40.

Heme iron from meat consumption appears to be a more specific culprit in the association between dietary iron and various cancers, as shown through an enhanced risk for gastrointestinal cancers in the Iowa Women's Health Study 41. Another noteworthy component of the Iowa Women's Health Study is the negative association between gastrointestinal tract cancers and zinc, a metal that was believed to be an antioxidant 41. In a more recent study, dietary iron consumption was shown to be associated with a higher risk for breast cancer in women; however, when antioxidant supplements were given in a SU.VI.MAX (from the French “Supplémentation en Vitamines et Minéraux Antioxydants”) trial, the association was no longer significant 42. Both of these studies suggest that the pro-oxidant activity of iron may be involved in cancer development.

Cancer in the colon and rectum is particularly associated with the consumption of red and processed meat. An analysis by Etemadi et al. found that total red meat and processed meat were strongly associated with colorectal cancer in all sites of the colon and rectum. Both meats had a greater associated risk than fish and poultry substitutes 43. Consumption of red meat is known to introduce other carcinogenic compounds to the body such as polycyclic aromatic hydrocarbons, heterocyclic amines (HCAs), N-nitroso compounds, and secondary bile acids, all of which are generated at various stages in the process of producing and cooking meat 44. Because these association-based studies could not account for other carcinogens as confounding factors, the mechanisms of red meat consumption in carcinogenesis have been harder to confirm. In the case of the Etemadi et al. analysis, red and processed meat are known to have more iron, but they are generally also known to have more total fat and saturated fat, which can cause changes in the gut microbiome and inflammatory status, placing people at a higher risk of cancer 45.

Bastide et al. demonstrated the effects of carcinogenic compounds from red meat *in vivo* and indicated heme iron as the most significant factor in tumor development as opposed to other potentially carcinogenic compounds from the meat 46. The group further identified a potential metabolic mechanism behind the observed effects. Their results indicate that the development of reactive aldehydes from heme iron metabolism may be a driving factor in tumor development. In the aforementioned study, HCAs presented at levels toxic to humans exhibited a surprisingly low effect on initiating intestinal carcinogenesis in rodents, an observation that the authors suggest may be related to differences in the xenobiotic metabolism of such compounds between the rodent models and the known effects on humans 46. Despite the study's limitation on identifying the intersecting effects of HCAs and heme on carcinogenesis, the more specific effects of heme iron alone suggest it to be a contributor to colorectal cancer development.

Diseases such as beta-thalassemia and hemochromatosis predispose individuals to developing cancer and other diseases later in life 47-50. Hereditary hemochromatosis is a genetic disorder that results in mutation-induced dysfunction of genes working as gatekeepers of iron circulation including hepcidin, its upstream regulator hemojuvelin, the iron-sensing transferrin receptor 2, and FPN. Hemochromatosis leads to an imbalance in iron homeostasis that results in iron overload and a range of other symptoms affecting various organs and systems 51. A study by Elmberg et al. reported that individuals with hereditary hemochromatosis exhibit an increased risk for developing cancer, particularly in the liver and primarily hepatocellular carcinoma as opposed to biliary tract related cancer 52. A significantly elevated risk to developing other cancers was not observed, likely due to the liver being one of the most affected tissues of iron overloading along with cardiac tissue and endocrine glands 53.

Beta-thalassemia is a group of genetic diseases marked by the common feature of improper beta chain synthesis in the hemoglobin protein of the blood. The featuring clinical presentation in thalassemia is anemia. The disease can range from major to minor classification, which corresponds with severe to asymptomatic or mild symptoms, respectively 54. The molecular physiology of beta-thalassemia presents an interesting case of both iron overload and iron deficiency, a phenomenon centered on hepcidin regulation 55. As previously discussed, hepcidin can prevent iron overload by cutting off recycling of iron. However, conditions of hypoxia and anemia can inhibit hepcidin in order to restore oxidation capacity (**Figure 2A**).

Patients with hemoglobin E beta-thalassemia, a severe form of the disease, were found to have impaired hepcidin function and higher TfR1 levels as a result of an increased erythropoietic drive stemming from the continuously failing erythropoiesis that is caused by improper hemoglobin production 56. Previously, the consequences of beta-thalassemia were life threatening, but as new treatments continue to extend the lifespans of these patients, the increased risk of cancer development has emerged, suggesting that the physiological connection may have been masked by early mortality. The iron overloading that occurs in individuals with beta-thalassemia, like with hemochromatosis, appears to be a risk factor for certain types of cancer, especially hepatocellular carcinoma 57.

Cigarette smoke is a known risk factor in the development of cancer, however a recent report indicates that smoking cigarettes may promote the development of lung cancer and other related pulmonary diseases through dysregulated iron metabolism, including iron deposition 58. Although cigarettes are known to contain many carcinogens, some even shared with red and processed meat, evidence suggests that changes in iron metabolism may precede the lung diseases 58, 59.

### Metabolic changes in iron-mediated carcinogenesis

Metabolic profiling of cancer cells enables scientists to better understand the implications of iron metabolism in cancer. An analysis of the metabolic profile of cholangiocarcinoma cells noted a strong shift towards iron retention in the cells, in agreement with the concept of iron dependence in cancer cells 60. The shift in cell metabolism towards iron accumulation also appears to play a role in the development of leukemia. Widespread dysregulation of iron metabolism in leukemia involves increased cellular iron import caused by overexpressed TfR1 and decreased iron export due to reduced expression of FPN 61. A study by Marques et al. noted a phenomenon of both iron acceptance by breast cancer cells, as well as iron donation by immune cells in the local tumor environment 62. One study shows that increased systemic hepcidin levels occurring as a result of a dysregulated hepcidin-FPN axis promotes breast cancer growth 63. Recently we found that ectopic hepcidin expression in colorectal cancer tissues is essential for maintaining cell proliferation due to the role of iron in both nucleotide synthesis and mitochondrial metabolism 64. Further implications of hepcidin-FPN axis regulation in cancer will be discussed later as a therapeutic avenue against cancer.

STEAP proteins can be overexpressed in a variety of cancers, including prostate, colon, ovarian, bladder, and pancreatic cancers 65. Despite the relevance of STEAP proteins in the progression of certain tumors, their effect is not universal. STEAP1 is overexpressed in gastric cancer cells and increases cell proliferation, migration, invasion and tumor growth 66. STEAP2 works inversely with breast cancer cell malignancy 67. When STEAP2 is overexpressed, it exhibits an inhibitory effect on proliferative pathways 67. STEAP3 overexpression helps cancer cells build caches of iron, leading to higher ferritin levels 68. This change in metabolism proved valuable for cell survival in the face of iron withdrawal 68. We found that intestine-specific overexpression of STEAP4, which is highly increased in colorectal cancer, increases mitochondrial iron accumulation, oxidative stress and susceptibility to colon tumors 69.

The altered iron homeostasis may be affected, in part, by IRPs, which exert post-transcriptional control over mRNAs, as evidenced by discrepancies between transcript levels and protein levels 70. IRP1 inhibition mediated reduced TfR1 expression is critical for the tumor suppressive role of Sirtuin 3 in pancreatic cancer cells 71. IRP2 was found to be responsible for changes in prostate cancer cells that resulted in greater cellular iron import, as well as having control over progression of the cell cycle 72. Moreover, IRP2 affects breast cancer growth through control of TfR1 and ferritin, with increased TfR1 resulting in an influx of cellular iron and an increase in the labile iron pool through ferritin downregulation 73.

In addition to IRPs, which affect mRNA transcripts, hypoxia signaling through the HIFs has been shown to exert control over gene transcription. HIF-2α is the major HIF isoform and regulates the expression of genes involved in iron metabolism. Moreover, HIF-2α appears to be overexpressed in colorectal and breast tumor cells and is associated with poorer prognosis 74, 75. In breast cancer, human epidermal growth factor receptor 2 augments HIF-2α expression 74. In colon tumors, HIF-2α is responsible for upregulating DMT-1 expression, resulting in a dysregulation of iron homeostasis thereby promoting cancer progression 76. HIF-2α may also be involved in the development of colon cancer in individuals with inflammatory bowel diseases by promoting the previously mentioned change in mitochondrial iron metabolism through direct upregulation of STEAP4 69.

With the pattern of iron loading in mind, the question moves its focus towards what the cells do with the extra iron. As mentioned before, iron is essential for various cellular functions including the processes necessary for energy production and replication [See *Iron Absorption and Recycling*]. Accumulating intracellular iron affects the cell cycle; we and others have seen that iron likely binds to cyclin dependent kinase 1 (CDK1), a major mitotic component, and activates it to trigger pro-proliferative downstream signaling 77, 78. CDK1 is overexpressed in colorectal cancer and predicts poor prognosis 79. Taken together, the body of studies on iron controlled CDK1 presents insight into one way that iron can manipulate the excessive growth characteristic of tumors. Furthermore, dysregulation of ribonucleotide reductase activity, essential for DNA synthesis, was found to result in carcinogenesis *in vivo* with particular concern on the M2 subunit overexpression being an accelerator of the malignant process 80.

Ni et al. reported that iron accumulated in the mitochondria through the upregulation of mitochondrial iron import proteins mitoferrin 1 and 2, and promoted a transformation of the cellular metabolism towards glycolysis known as the Warburg effect 81. The Warburg effect was discovered in the early 20^th^ century and is thought to be an adaptation that simplifies energy production in exchange for fast-tracking synthesis of various biomolecules 82. An iron chelator prevented the Warburg effect, indicating the bona fide role of iron in cellular metabolic reprogramming 81. Mitochondrial iron can be repurposed through the process of mitophagy, a selective form of autophagy that removes damaged mitochondria. This process can help tumor cells survive and adapt, but it may not be entirely beneficial to cancer cells either. For example, we have recently shown that PTEN-induced kinase 1-dependent mitophagy is essential for suppressing colon tumor growth 83. This is consistent with a previous report showing that increases in degradation of iron-rich mitochondria by mitophagy in intestinal epithelial cells cause an excess of iron buildup in lysosomes, resulting in increases in ROS produced by the Fenton reaction 84. Subsequent addition of the lysosomal membrane permeabilization inducer chloroquine raises the pH of the lysosomes and compromises the integrity of the lysosomal membrane, resulting in an increased leakage of cathepsins, a group of proteases, into the cytosol and alters cellular antigen presentation to elicit cytotoxic T-cell immune responses 84. Thus, lysosomal iron accumulation can be exploited to induce anti-tumor immunity and restrict tumor growth.

In relation to oxidative stress, the accumulation of iron in cancer cells does bring the great dangers to the cells in addition to its benefits. In other words, cancer cells are no exception to the rules of iron; however, they develop ways to stay ahead. The glutamate-cystine antiporter, also known as system Xc-, plays a vital role in the antioxidant defense system of cells by supplying cystine to the cell 85; the amino acid regenerates glutathione, which terminates ROS through glutathione peroxidase 4 (GPX4) 86. The system Xc- component xCT (light chain) encoded by the gene SLC7A11 is overexpressed in non-small cell lung cancer cells 87. High expression of SLC7A11 is associated with poorer outcomes and may contribute to metabolic reprogramming in tumors 87. In our lab, we found that hemin, a heme derivative containing a porphyrin-bound iron, may promote colorectal tumor cell survival and growth through induction of sestrin2, a protein that counters oxidative stress 88. Sestrin2 overexpression noticeably prevented regulated cell death processes and resulted in larger tumors when accompanied with increased iron via hemin, whereas sestrin2 overexpression without excessive iron maintains its tumor suppressive properties 88. This conditional duality of sestrin2 highlights the importance of iron homeostasis in cancer by demonstrating a mechanism of action in which heme iron dysregulation actively promotes tumor development.

In summary, iron dysregulation may come in many forms through different sources, and cancer cells undergo alterations in their gene expressions to favor the import and retention of iron leading to larger supplies to sustain their rapid growth characteristics. These transformations may be regulated at various points and affect the biosynthetic and proliferative statuses of the cell. In order to avoid cellular damage from iron toxicity, cancer cells take advantage of antioxidant systems. Understanding iron toxicity, iron-dependency and addiction, and homeostatic disruption, including the various upstream controls and downstream effectors of the process, has become an increasingly popular area of study in terms of cancer treatment. The following section discusses the potential of targeting iron metabolism for therapeutic purposes in further detail.

## Potential Cancer Therapeutic Targets and Biochemical Treatments

**Figure 3:**
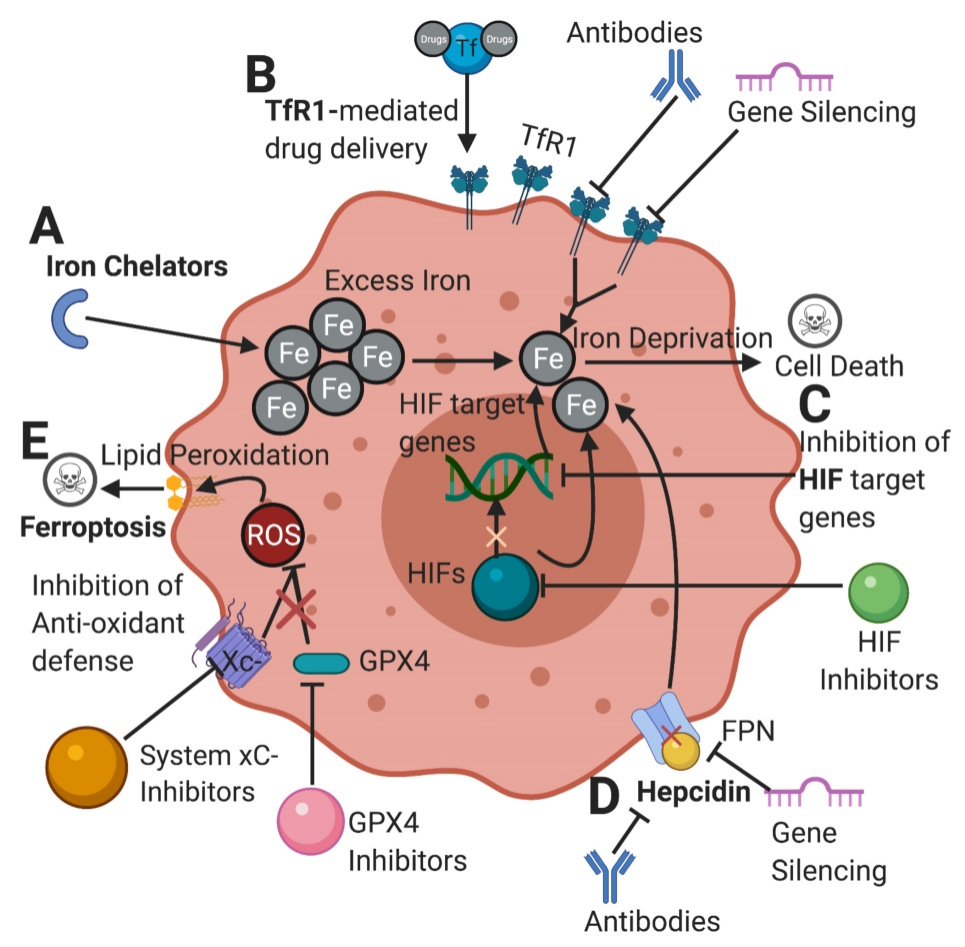
** Potential therapeutic pathways in cancer targeting abnormal iron metabolism. [A]** Iron chelators limit the available amount of iron in tumor cells. Chelators and other drugs can be delivered through a TfR1-mediated drug delivery. [**B**] Inhibiting TfR1 by antibodies and gene silencing can lower iron import, depriving the cell of its desired iron content. [**C**] Inhibition of HIFs and their target genes can ultimately limit the amount of iron available for cells and hinder the ability of cancer cells to proliferate. [**D**] Inhibition of hepcidin-FPN axis can increase cellular iron export depriving the cell of iron. [**E**] Inhibition of cellular antioxidant defenses such as system xC- and GPX4 renders the cell prone to ROS accumulation from iron metabolism, leading to lipid peroxidation and ferroptosis. TfR1: transferrin receptor; HIFs: hypoxia-inducible factors; FPN: ferroportin; xC-: cystine-glutamate antiporter; GPX4: glutathione peroxidase 4; ROS: reactive oxygen species. Created with BioRender.com

Due to the critical role of iron in cancer, targeting iron metabolism emerges as a novel therapeutic strategy in the treatment of cancer. Here we have chosen to highlight and discuss several promising strategies that are currently being explored in the world of cancer therapeutics (**Figure 3**).

### Iron Chelation

Iron chelation is a developing strategy aimed at sequestering iron from usage in tumor cells (**Figure 3A**). Iron chelators had been used extensively to treat disorders of iron-overload in order to help patients evade the effects of iron toxicity 89. Deferoxamine (DFO), deferiprione (DFP), and deferasirox (DFX) are three chelators commonly used in clinical settings. However, there are varying degrees of toxicity with them as well 90. DFO treatment of breast cancer cell lines MCF-7 and MDA-MB-231 resulted in significant reduction of the intracellular iron supply and decreased cellular regeneration and survival 91. Another study of DFO using breast cancer cell lines showed strong results in combining chelation with radiation treatment, which resulted in increased tumor cell death 92. DFP also exhibits strength as a chelator with an ability to limit tumor growth, migration, and metabolism 93. DFP was found to inhibit overall cellular respiration capacity corresponding positively with the increase of dosage while generating ROS in the same pattern 94. DFX was shown to inhibit cell cycle progression, while downregulating proliferative pathways in gastric cancer cells 95.

In addition to DFO and its derivatives, other compounds with chelating abilities have gained attention with regard to cancer treatment. Shang et al. demonstrated that the chelators ciclopirox olamine and Di-2-pyridylketone-4,4-dimethyl-3-thiosemicarbazone (Dp44mT) could effectively permeate cancer cells and inhibit proliferative signaling through the mammalian target of rapamycin pathway 96. Dp44mT is a newer chelator that has also been studied against a number of cancer cell types in controlled experimental trials. Using Dp44mT against osteosarcoma cells *in vitro* and *in vivo*, Li et al showed that the chelator was able to inhibit cancer cell vitality and proliferation likely through caspase-dependent apoptosis 97. *In vivo* xenograft transplantation resulted in an average decrease in tumor mass of 62.22% in Dp44mT treated mice at the 30-day sacrifice point 97. Moreover, Krishan et al. found that Dp44mT was able to disrupt metabolic processes in order to drain ATP levels to the point of AMP-activated protein kinase activation, which eventually leads to autophagy/catabolism 98.

Two novel chelators, known as the super-polyphenols 6 and 10, were observed by Ohara et al. to have anticancer properties comparable to DFO and DFX as evidenced by induced apoptotic activity 99. Notably, these beneficial effects came without the associated additional toxicity of DFO and DFX 99. More studies using the novel super-polyphenol chelators are needed to reinforce the results from Ohara et al., but the study appears to be in line with established anticancer properties of chelation. Curcumin has also been reported to have iron-chelating properties and was used in a recent study to determine its effects on caspase-dependent apoptosis. Results indicated that curcumin was indeed effective at chelating iron and inducing apoptosis as evidenced by the expression of apoptosis markers caspase-3 and caspase-9. However, there were also protective actions taken by the cells in order to counter the effects of curcumin 100.

A potential drawback in chelation therapy may be the unabated attempts of cancer cells to restore iron homeostasis. Chen et al. reported that the usage of DFO against breast cancer cells resulted in increased expression of the iron import proteins DMT1 and TfR1, leading to an overall increase of intracellular iron concentrations 101. This phenomenon was only observed in aggressive triple-negative breast cancer (TNBC) cell lines, while the estrogen receptor+ non-aggressive cell lines did not experience the same phenomenon of iron accumulation. Activation of the IL-6/phosphatidylinositol-3 kinase (PI3K)/ protein kinase B (PKB, also known as Akt) pathway was observed in DFO treated cells from the aggressive TNBC groups in contrast to the estrogen receptor+ positive groups 101. The IL-6/PI3K/Akt pathway is an inflammatory pathway that promotes cancer cell survival 102. IL-6/PI3K/Akt pathway regulated iron uptake protein expression is suspected to be responsible for iron accumulation in TNBC cell lines 101; however, the underlying reason for the exclusivity to TNBC cells is not clear.

In a study by Liu et al., DFO promoted cell viability through a different mechanism, HIF-1α, which is likely to have arisen as a consequence of an iron deficient state 103. Although these studies were performed *in vitro*, they present possible concerns over the potential side effects of a more general treatment such as the sequestration of iron as opposed to more specific pathway targeting methods. The ability of cells to adapt to their environment poses particular challenges for anticancer treatments. A possible counter to this may be through the use of combined chelation and other therapies. In an *in vivo* study by Lang et al. involving administration of the chelator DFO and a HIF-1α inhibitor known as Lificiguat (YC1), anticancer efficacy was compared between pancreatic cancer cells treated with and without YC1. Compared to chelation treatment alone, use of DFO with YC1 was more effective in destroying cells and inhibiting their ability to overcome the lack of iron 104. Resistance to therapy is not exclusive to chelators. The common chemotherapy agent cisplatin is met with resistance that can actually be overcome with the help of chelators 95, 105. In addition to countering HIF-mediated resistance, combining chelators with other conventional chemotherapeutics has gained attention for studies in both basic science and clinical investigation to counter other forms of resistance and amplifying the effects of other drugs (**Table 1**).

Iron chelators DFO and DFX have been reported to synergize with the pyrimidine analog 5-Fluorouracil (5-FU), a classic chemotherapy drug that disrupts DNA synthesis, to diminish esophageal cancer growth *in vitro* and *in vivo* 105. DFP and Dp44mT can synergize with 5-FU to treat glioblastoma 106 and breast cancer 107, respectively. DFO was reported to synergize with platinum-based chemotherapy drugs in different cancer types including ovarian cancer 108, neuroblastoma 109, cervical cancer 110. DFX, Triapine and Dp44mT were able to synergize with cisplatin to treat TNBC 111, advanced-stage solid tumor malignancies 112 and cisplatin acquired resistant lung cancer 113, respectively.

Chelation has been shown to synergize with inhibitors of topoisomerases 114-118, which are involved in DNA strand opening during replication, as well as inhibitors of poly-ADP-ribose polymerase (PARP) 116, which is involved in DNA repair. The chelator triapine was able to suppress the growth of BReast CAncer gene-wild type and PARP inhibitor-resistant ovarian cancer cells 119. DFP enhanced the anti-cancer efficacy of the alkylating agent temozolomide that damages DNA 120. Tury et al. demonstrated that DFX can work successfully in combination with the alkylating agent cyclophosphamide against TNBCs in mouse models 111. Iron chelation has also displayed synergistic activity with the intercalating agent doxorubicin in both leukemia 121 and solid tumors 107, 111, 122.

Combining chelators with radiation therapy has been shown to amplify the anti-cancer effects of radiation therapy 123-126. The mechanism of action has been suggested to result from an inability to recover from radiation damage due to a lack of iron 123, 125. In several studies Kunos and colleagues investigated and discussed clinical applications of triapine in combination with radiochemotherapy for the treatment of gynecological cancers, namely cervical and vaginal cancers 127-130. One study showed that triapine in addition to radiochemotherapy produced a progression-free survival rate at 18 months of 67% as opposed to 25% without triapine 128. Another study observed a 3-year disease free survival rate of 80 percent, and an overall survival rate of 82 percent in cervical cancer patients 127. Comparing patients who received triapine in addition to cisplatin-radiotherapy as opposed to the radiochemotherapy without triapine showed a 15 percent difference in a 3-year estimated survival rate (92 vs 77, respectively) 130.

In the aforementioned cases, the chelator or the complementary drug appears to make up where the other comes up short. The delivery design previously discussed in the study by Lang et al. also took advantage of another characteristic of cancer 104, TfR1 overexpression, which leads to the next section.

### TfR1

Targeting TfR1 is developing into a promising avenue of cancer treatment (**Figure 3B**). This is rooted in the elevation of TfR1 expression in tumor cells as compared to normal cells 131. Though there are greater levels of TfR1 in cancer cells, there are differences in TfR1 expression in normal cells depending on the tissue and cell type. For example, normal kidney, adrenal gland, and liver cells express TfR1 in greater amounts 132. As such, TfR1 targeted cancer therapeutics could potentially cause adverse side effects in healthy tissues and possibly larger organ systems. Nevertheless, increased TfR1 expression is associated with the progression of disease in differing types of cancer cells, including renal cell carcinoma (RCC) and esophageal squamous cell carcinoma, making it a strong target for treatment 132, 133.

A clearer understanding of the upstream mediators regulating TfR1 will be important for finding treatments centered around it, so as to avoid unwanted secondary effects. The oncogene c-Myc is known to be a potent regulator of proteins involved in proliferation and was previously determined to affect the expression of TfR1 134. Expression of c-Myc is dependent on circadian fluctuations 135. This fluctuation affects the internalization of antitumor drugs that rely on TfR1 for entry into the cell. A paper by Okazaki et al. noted that the effects of a platinum-containing antitumor drug were strongest when administered around times when c-Myc and TfR1 were at higher levels as evidenced by greater tumor reduction 135. Furthermore, IRP2 may play a role in the circadian pattern of TfR1 protein expression 136. Findings from these studies suggest that time may be a factor in determining the outcomes of TfR1-involved cancer treatments, however additional studies are needed regarding the circadian clock effect and anticancer therapeutics.

TfR1 has also been shown to be an effective binding site for the delivery of H-ferritin nanocarrier, which can be loaded with anticancer drugs. This method of drug delivery yielded favorable results in gastric cancer and may be able to take advantage of the overexpression of TfR1 in other types of cancers for a more accurate delivery of drugs 137. Targeted degradation of TfR1 may also be a potential avenue of therapy. A test of anti-TfR1 monoclonal antibodies demonstrated a method of accurately targeting the receptor in cancer cells 138. While this is one possible method of exploiting TfR1, there are noted risks of adverse immune reactions in general with the usage of monoclonal antibodies 139. Using short-interfering RNA (siRNA) to silence TfR1 was demonstrated to be effective in both silencing TfR1 gene expression and in promoting apoptotic cell death 140. Use of siRNAs for anticancer therapeutics is mostly in a developing stage and has specificity advantages over both antibodies and drugs, but it also has the potential for drawbacks such as interference of other genes and enzymatic degradation 141. More studies are needed for both monoclonal antibodies and gene silencing, but they exist as possibilities in cancer treatment. Along with receptor targeted delivery of drugs, monoclonal antibodies and siRNA-based therapeutics keep the door open to either exploit or inhibit the effects of the TfR1-cancer association.

### HIF

HIFs exist to counteract the environmental changes in a cell that result in a perceived state of hypoxia 142 (**Figure 3C**). In addition to the previously discussed increases in cellular iron content mediated by HIF-2α [See *Metabolic changes in iron-mediated carcinogenesis*], their power over cellular transformation extends further, which makes them a valuable target. HIF-2α may play a role in immune system evasion by tumors through the upregulation of cyclooxygenase-2 (COX2), resulting in a downstream increased production of prostaglandin E2 (PGE2), a molecule with immunosuppressive activities in the COX2/microsomal prostaglandin E synthase-1/PGE2 pathway 143, 144. As previously mentioned, activation of the HIF pathways is a crucial adaptation mechanism for the continued survival of tumor cells and may arise as an obstacle in chelation treatment if unaccounted for. Therefore, research into methods of inhibiting HIF activation constitute an important area in developing treatments for cancer.

A number of drugs are currently being studied for their ability to inhibit the HIF pathways. These drugs use a variety of mechanisms including transcriptional and translational inhibition, inhibition of quaternary structure formation, and promotion of degradation 145. A review of tested HIF inhibitors identifies and summarizes the observed effects of HIF-1α and/or HIF-2α inhibitors and details their mechanisms of action 146. One of the inhibitors from this review was YC-1, which was used in a chelation therapy study as a means of inhibiting HIF-1α activity and enhancing iron chelation 104. This was one of several mentioned inhibitors that targeted transcriptional activity. Several other inhibitors reviewed were reported to target molecular chaperones such as heat shock protein 90, a process that can negatively impact HIF-1α and HIF-2α stability 146. Usage of the HIF inhibitor TAT-ODD-procaspase 3 (TOP3) was also shown to yield success in murine models of pancreatic cancer when combined with gemcitabine or TS-1, extending survival rates from 0 to 25% after 100 and 50 days for TOP3-gemcitabine and TOP3-TS-1 combinations, respectively 147.

The search for other potential inhibitors of HIF and its effective pathway can open more doors for therapeutics as well. The gene *Parkin*, known for its role in Parkinson's Disease, has been reported to have tumor suppressing capabilities, and increases in *Parkin* expression in mice models resulted in degradation of HIF-1α and resultant inhibition of tumor progression, possibly through a ubiquitination-degradation mechanism 148. Delivery of a modified, soluble therapeutic form of the protein into Parkinson's Disease model rodents successfully protected against advancement of Parkinson's disease 149. This may suggest that the protein could have therapeutic potential if it can maintain function with modifications for drug delivery.

With regard to downstream targets of HIF, HIF-2α was found to indirectly regulate Yes-associated protein 1 (YAP1), a key mediator of the Hippo pathway, which is known to regulate the growth and regeneration processes of several organs, including the intestines 150, 151. The exact mechanism by which HIF-2α regulates YAP1 needs further investigation, but it is one way that HIF activity can promote the growth of tumors 150. Vascular endothelial growth factor-A (VEGF-A) is another downstream target of HIF and the major driver of angiogenesis 152. A study by Lu et al. demonstrated that micro-RNA miR-140-5p exerts an inhibitory control over VEGF-A expression 153. Further studies on nucleic acid-based inhibition as a whole could shed light on a potential field of gene therapy targeted at VEGF. Bevacizumab, a humanized anti-VEGF monoclonal antibody is a first-line treatment for metastatic colorectal cancer in combination with chemotherapy 154. Several other anti-VEGF molecular inhibitors have been developed and studied, demonstrating an ability to maintain tumor specific effects 155.

Overall, the network of HIF proteins is complex, but it is a critical part of cancer development. HIF-based therapy can either target the proteins themselves or key points in the signaling pathways. The above-mentioned papers have demonstrated the therapeutic efficacy of targeting HIF at various points in associated signaling pathways, thus this field of study has recently gained the interest of a number of researchers.

### Hepcidin-FPN

Targeting the hepcidin-FPN system stands out as a possible option in mitigating the effects of iron overload on tumor progression (**Figure 3D**). As previously mentioned, hepcidin promotes cellular retention of iron by acting as an inhibitor of FPN (see Iron Homeostasis). Decreased FPN and increased intracellular iron results in greater proliferation of cancer cells as evidenced in myeloma studies 156, 157. Serum levels of hepcidin may also serve as a prognosis indicator in cancer cases 156, 157. Traeger et al. observed a positive correlation between hepcidin and growth/differentiation factor-15, a proliferative indicator in upper urinary tract urothelial carcinoma and RCC. The authors observed elevated levels of hepcidin in cohort subjects with metastatic upper urinary tract urothelial carcinoma and RCC relative to their non-metastatic counterparts, as well as non-cancer bearing control subjects 158. However, there was a multicenter epidemiological study showing an inverse relationship between hepcidin and gastric cancer 159. In this study, the authors propose that the observed lower levels of hepcidin may be due to systemic blood loss in earlier stages of the disease 159. It must be noted that not all cancers elicit the same changes in serum hepcidin. A comprehensive review on hepcidin regulation highlights the observed differences in hepcidin expression in various cancerous tissue types 160. Most cancers exhibited the expected hepcidin production pattern, with elevated levels of local and systemic hepcidin, while liver carcinoma went against the grain with decreases in both local and systemic hepcidin 160. The exact mechanism of this phenomenon is unclear, but it has been proposed that the suppression of hepcidin expression in liver cancer may be the body's attempt to ultimately increase the total amount of available iron by allowing increased efflux from duodenal enterocytes 161. Further studies are needed to determine if this is the case and to delineate the downstream effects of abrogated hepcidin production.

As the general consensus points towards increased hepcidin being linked with progression of disease state, various ways to inhibit hepcidin are being studied. Angelica Sinensis Polysaccharide, used in East Asian medicine and food, has the ability to inhibit hepcidin and reduce intracellular iron concentrations, including in tumor cells 162. Angelica Sinensis Polysaccharide decreased levels of hepcidin by 54.55% in 4T1 breast tumor bearing mice and 47.29% in H22 hepatocellular carcinoma bearing mice 162. Another paper investigating the role of hepcidin by Vadan-Raj et al. studied the efficacy of hepcidin monoclonal antibody LY2787106 in human patients with cancer-associated anemia. The results from this study indicated a successful effect of the monoclonal antibody LY2787106, as evidenced by increased serum iron levels. Another outcome in favor of monoclonal antibody LY2787106 was the relative tolerability by the patients. The major drawback of the treatment was that the results were short lived with serum iron levels returning to pre-treatment levels within 8 days for reasons that are still only speculated 163. The authors suggest that the neutralization of hepcidin may not be sufficient on its own. In another study, Torti et al. employed an anti-hemojuvelin antibody complex in order to inhibit hepcidin synthesis. This method was effective at decreasing hepcidin synthesis and lowering iron in the liver, but it failed to successfully lower iron in liver tumors 164. The authors propose that this phenomenon may be explained by the resulting increase of systemic iron in the circulation. It is possible that inhibition of hepcidin is not a standalone option for treating cancers, but due to its important role in iron homeostasis, it should not be ignored in the discussion of integrated treatments.

### Ferroptosis

Regulated cell death can proceed through several avenues, including a recently identified mechanism known as ferroptosis **(Figure 3E)**. The field of ferroptosis therapeutics has gained more interest in recent years since its official notation in 2012 165. Understanding the pathways and mechanisms involved in ferroptosis is key to developing it as a reliable area of treatment. Independent of the common mechanisms found in the caspase-dependent forms of cell death, ferroptosis proceeds through an iron-dependent process involving ROS overload that leads to membrane lipid peroxidation and compromised structural integrity of the cell 166. Given the well-established connections between iron and cancer, it is a useful and somewhat paradoxical strategy to use iron to treat cancerous cells. Iron oxide nanoparticles such as ferumoxytol [Feraheme®] have been approved by the FDA. Their potential use for diagnosis and treatment of cancer is very attractive. A recent study showed that cisplatin loaded iron oxide nanoparticles can induce ferroptosis via accelerated Fenton reaction to inhibit orthotopic brain tumor growth 167. Iron oxide nanoparticles can also synergize with heat stress to produce overwhelming levels of lipid peroxides, and consequently sensitize the tumor to ferroptosis 168.

Cellular antioxidant defense systems appear to make up a central point in understanding and developing the potential of ferroptosis. T-cells may naturally induce ferroptosis in tumors through the use of the cytokine interferon gamma to disrupt the cystine import system 169. Erastin, the drug used in the 2012 study that led to the official notion of ferroptosis, is known to disrupt system Xc- 162. However, erastin is not suitable for *in vivo* use due to undesired pharmacological properties 170. Moreover, Nedd4 has been shown to be a potential counter to erastin-induced ferroptosis as it leads to the degradation of the mitochondrial voltage dependent anion channels 2 and 3, on which erastin treatment is dependent 171. Efficacy of erastin may be recovered through the use of Nedd4 inhibitors, however further studies are needed to determine clinical applicability in overcoming drug-resistance in tumors 172.

Targeting cellular antioxidant defense through the use of siRNA to silence SLC7A11 expression needed for cysteine import resulted in decreased cancer cell vitality *in vitro* and *in vivo* 173. It has been found that transcription factors Ets proto-oncogene 1 (Ets-1) and activation transcription factor 4 may work in tandem to enhance transcription of SLC7A11 in response to oxidative stress 174. On the other hand, activation transcription factor 3 was found to inhibit transcription of SLC7A11 independent of the tumor suppressor gene p53 175. These controls could serve as a way forward in gene-based therapy for either inhibition or activation. So far, no study has examined the effects of such actions on these genes in regard to ferroptosis. Sulfasalazine, an anti-inflammatory and anti-rheumatic drug, has an inhibitory effect on xCT and was shown to cause a reduction in oxidative stress damage repair 176.

Treatment of cells with Ras-selective-lethal 3 was shown to enhance ferroptosis through GPX4 inhibition and subsequent increase in iron intake via transferrin 177. In the event of drug resistance, silencing the nuclear factor erythroid 2-related 2 gene, which is a part of a ferroptosis resistance pathway, can restore the efficacy of Ras-selective-lethal 3 178. GPX4 can also be inhibited by small molecules such as ML-210, which works through a strong covalent binding of a selenocysteine residue in the protein 179. Ferritinophagy, a process of autophagic degradation of ferritin mediated by nuclear receptor coactivator 4 180, can also induce ferroptosis in cancer cells 181. The generation of ROS by erastin treatment has been shown to achieve the autophagy necessary to degrade the iron-containing proteins like ferritin in order to achieve the intracellular overload of iron that helps lead to ferroptosis 182.

Prominin2 can also drive ferroptosis resistance in breast cancer cell lines through the exocytotic removal of cellular iron and alleviation of oxidative stress 183. Results from this study showed that ferritin co-localized with prominin 2 and congregated in the exosomes. Interestingly, MDA-MB-231 cells, previously discussed in the chelation section for their affinity for iron in the face of pharmacological adversity, exhibited higher sensitivity to GPX4 inhibition-induced oxidative stress and ferroptosis as a result of lower prominin 2 levels. Supplementation of prominin 2 to cells generated resistance to GPX4 inhibition 183. The previously discussed FPN has its own implications in the context of ferroptosis sensitivity and resistance. Geng et al. demonstrated that siRNA knockdown of FPN increased the sensitivity of neuroblastoma cells to erastin-induced ferroptosis compared to erastin treatment alone 184. Lipid peroxidation measured by flow cytometry was significantly higher in FPN knockdown cells than control cells after erastin treatment. Furthermore, the incubation of cells with the FPN inducer ponasterone resulted in enhanced viability of cells co-treated with erastin, and enhanced viability was diminished when cells were transfected with FPN siRNA 184.

In addition to antioxidant and iron related metabolism, ferroptosis is also dependent on the metabolism of lipids. Lipid metabolism may also provide a potential avenue for targeting ferroptosis. Acyl-CoA Synthetase Long Chain Family Member 4 (ACSL4) used in phospholipid synthesis, has been implicated as favorable to ferroptosis. Both knockout and pharmacological inhibition of its activity are able to impede ferroptosis 185. In a study by Doll et al., it was suggested that ACSL4 activity promotes ferroptosis as a result of the preference for long poly-unsaturated fatty acids (PUFAs) as a substrate, in particular omega-6 fatty acids. This was based on the exogenous supplementation of omega-6 presenting greater vulnerability to ferroptosis than omega-3 185. In contrast to PUFAs, monounsaturated fatty acids (MUFAs) present a lower susceptibility to ferroptosis. Magtanong et al. demonstrated that supplementation of MUFAs in cells led to a restructuring of plasma membrane lipid composition that supplanted pre-existing PUFAs with MUFAs and lowered sensitivity of cells to ferroptosis 186. The MUFAs did not display the susceptibility to oxidation at the 11^th^ carbon, a marker of ferroptotic lipid peroxidation, allowing membrane integrity to be preserved 186.

Many of the treatment concepts related to ferroptosis are still in a relatively nascent stage, and more studies are needed to eventually translate them into effective clinical application. Ferroptosis remains a promising field of anticancer therapeutics with multiple avenues for success as evidenced by the results in studies targeting system Xc-, GPX4, and ferritinophagy, as well as ROS overloading. One of the key challenges in targeting iron for cancer therapeutics is that iron is tightly regulated and an essential component to cellular function. Attempts to manipulate iron levels in the cell have manifested in various forms of cellular resistance to ferroptosis and thus uncontrolled cellular proliferation. Additional studies on the pathways surrounding iron metabolism, antioxidants, and lipid metabolism are essential to better harness the potential of this emerging and challenging frontier in cancer therapeutics.

## Summary and Conclusion

Given the complexity of cancer, it is beneficial to find patterns within it in order to both prevent and treat the disease. Patterns of iron dependence in cancers open the door to a field of therapeutics that aims to target cancer cells with a greater specificity in order to both maximize therapeutic efficacy and avoid harm to healthy cells. Furthermore, understanding iron's nature as a pro-oxidant and its potential role as a collaborator in carcinogenesis may help to provide easy, beneficial preventative measures against cancer. While there are a number of studies discussed in this review that illustrate a connection between iron and cancer, there has yet to be a definitive mechanism of action elucidated. It is possible that there may not be a single route towards disease when it comes to iron and cancer. The links between consumption of certain meats and the development of cancer highlight this point: although the risk of cancer increases with consumption of more iron-heavy meat (e.g., red and processed meat), the meat also harbors many other known carcinogens, making the details of the connection often unclear. In carcinogenesis, iron may lead to oxidative stress with its redox reactivity and fuel the cancer stem cells with proliferative supplies, which has been illustrated in a recent review 187. This could serve as the basis of future studies in the matter.

Cellular iron metabolism has many individual components that make up a very delicate system that ensures vitality by providing the essential nutrients and, inadvertently, potential toxicity. Understanding these pathways has enabled scientific knowledge to advance to the point of identifying the extent of metabolic modification and dysregulation that occur in cancer and knowing how to use it. Targeting TfR1 can be useful as increasing levels of the receptor enhances cellular import of iron or other TfR1 ligand-conjugated anticancer drugs. Increased hepcidin levels resulting in greater intracellular iron concentrations is the basis for hepcidin based treatment that could inhibit or degrade hepcidin and relieve the iron accumulation. Studies on hepcidin inhibition show potential, but they also suggest that it may not be a standalone option. Sequestering iron by use of chelators is a more heavily tested method of targeting iron in general by depriving cancer cells of the iron that they need for their rapid growth. Cells may counteract the lack of iron through induction of HIFs. HIF inhibition may be an effective complement to iron chelation and other methods of therapeutics by severing a useful lifeline in iron and/or oxygen lacking cells, but further studies of treatment combinations are needed to better illustrate the effects of various methods on cancer cells. Regulated cell death by means of ferroptosis is a promising way of using iron against cancer cells 188. Unlike chelation and other treatments that decrease the level of iron in cells, ferroptosis needs iron in the cells to unleash its toxicity. Therapeutic methods that can induce this phenomenon may target the vital antioxidant defenses of the cell, which then gives way to the ROS-led destructive processes. A recent review has summarized the currently available pharmacological agents targeting iron metabolism 189. Keeping an eye on naturally occurring activation and inhibition mechanisms from transcription to post-translation can help shape and guide future strategies. *In vivo* testing and clinical trials will be necessary to generate a clearer image of the true potential of these treatments.

## Figures and Tables

**Table 1:** Iron chelation and conventional chemo-/radio-therapeutics

	Deferoxamine	Deferiprone	Deferasirox	Triapine	Dp44mT
**Antimetabolites**	5-FU	5-FU	5-FU	No report	5-FU
	two oesophageal adenocarcinoma cell lines, OE33 and OE19, and the squamous oesophageal cell line, OE21 105.	glioblastoma 106	two oesophageal adenocarcinoma cell lines, OE33 and OE19, and the squamous oesophageal cell line, OE21 105		MCF-7 breast cancer cells 107
	
	
	
	
	
	
					
**Platinum**	Cisplatin, Carboplatin, Oxaliplatin	No report	Cisplatin, Carboplatin	Cisplatin	Cisplatin
	ovarian cancer 108, neuroblastoma 109, human cervical cancer cells 110		triple-negative breast cancers 111	advanced- stage solid tumor malignancies 112	cisplatin sensitive and acquired resistant lung cancer cell lines 113
	
	
	
	
	
					
**Topoisomerase**	Etoposide, Irinotecan	No report	No report	Etoposide, Irinotecan	Etoposide
**inhibitor**					
	human neuroblastoma (NB) cell lines 114, human slice cultures of gastric cancer 115			epithelial ovarian cancer 116, patients with refractory solid tumors 117	three pediatric tumor cell- types, namely osteosarcoma (Saos-2), medulloblastoma (Daoy) and neuroblastoma (SH-SY5Y) 118
		
		
		
		
		
		
		
		
					
**PAPR inhibitor**	No report	No report	No report	Olaparib ovarian cancer [116, 119]	No report
				
				
					
**Alkylating agent**	CP	TMZ	CP	No report	TMZ, CP
	Neuroblastoma 109	Glioblastoma 120	triple-negative breast cancers 111		Pediatric tumors 118, MCF-7 breast cancer cells 107
**Intercalating agent**	Dox	No report	Dox	Dox	Dox
	Transformed cells from a murine PTEN-deficient T-cell lymphoma model and from T-cell acute lymphoblastic leukemia/ lymphoma (T-ALL/T-LL) cell lines 121		triple-negative breast cancers 111	patients with advanced solid tumors 122	MCF-7 breast cancer cells 107
					
Radiation	human hepatoma cell lines (HepG2 and PLC/PRF/5) 123	Chinese hamster V79 cells, human lung cancer cells 124	No report	three human tumor cell lines were evaluated: U251 (glioma), PSN1 (pancreatic carcinoma), and DU145 (prostate carcinoma) 125	a chemo- and radio-resistant cell line (PC-3) of prostatic cancer origin 126
					
**Radiochemotherapy**	No report	No report	No report	Patients with Stage IB2-IIIB Cervical Cancer, Advanced- Stage Cervical and Vaginal Cancers 127-130	No report
					

5-FU: 5-Fluorouracil; CP: Cyclophosphamide; Dox: Doxorubicin; TMZ: Temozolomide.

## Abbreviations

5-FU: 5-Fluorouracil
ACSL4: Acyl-CoA Synthetase Long Chain Family Member 4
CDK1: cyclin dependent kinase 1
COX2: cyclooxygenase-2
CP: Cyclophosphamide
DcytB: duodenal cytochrome B
DFO: Deferoxamine
DFP: deferiprione
DFX: deferasirox
DMT-1: divalent metal transporter 1
Dox: Doxorubicin
Dp44mT: Di-2-pyridylketone-4,4-dimethyl-3-thiosemicarbazone
FPN: ferroportin
GPX4: glutathione peroxidase 4
HEPH: hephaestin
HCAs: heterocyclic amines
HIF: hypoxia-inducible factor
IL-6: interleukin-6
IRE: iron response element
IRP: iron response protein
mRNA: messenger RNA
MUFAs: monounsaturated fatty acids
PARP: poly-ADP-ribose polymerase
PCBP: poly-r[C]-binding protein
PGE2: prostaglandin E2
PI3K: phosphatidylinositol-3 kinase
PUFAs: poly-unsaturated fatty acids
RCC: renal cell carcinoma
ROS: reactive oxygen species
siRNA: short-interfering RNA
STEAP: six transmembrane epithelial antigen of the prostate
Tf: transferrin
TfR1: transferrin receptor 1
TMZ: Temozolomide
TNBC: triple-negative breast cancer
TOP3: TAT-ODD-procaspase 3
UTR: untranslated region
VEGF-A: vascular endothelial growth factor-A
xC-: cystine-glutamate antiporter
YAP1: Yes-associated protein 1
